# Efficient DNA Coding Algorithm for Polymerase Chain Reaction Amplification Information Retrieval

**DOI:** 10.3390/ijms25126449

**Published:** 2024-06-11

**Authors:** Qing Wang, Shufang Zhang, Yuhui Li

**Affiliations:** School of Electrical Automation and Information Engineering, Tianjin University, Tianjin 300072, China

**Keywords:** DNA storage, DNA coding, biological constraint, nonspecific pairing constraint, storage density, ECA-PCRAIR

## Abstract

Polymerase Chain Reaction (PCR) amplification is widely used for retrieving information from DNA storage. During the PCR amplification process, nonspecific pairing between the 3’ end of the primer and the DNA sequence can cause cross-talk in the amplification reaction, leading to the generation of interfering sequences and reduced amplification accuracy. To address this issue, we propose an efficient coding algorithm for PCR amplification information retrieval (ECA-PCRAIR). This algorithm employs variable-length scanning and pruning optimization to construct a codebook that maximizes storage density while satisfying traditional biological constraints. Subsequently, a codeword search tree is constructed based on the primer library to optimize the codebook, and a variable-length interleaver is used for constraint detection and correction, thereby minimizing the likelihood of nonspecific pairing. Experimental results demonstrate that ECA-PCRAIR can reduce the probability of nonspecific pairing between the 3’ end of the primer and the DNA sequence to 2–25%, enhancing the robustness of the DNA sequences. Additionally, ECA-PCRAIR achieves a storage density of 2.14–3.67 bits per nucleotide (bits/nt), significantly improving storage capacity.

## 1. Introduction

With the rapid advancement of computer technology, efficiently storing vast amounts of information has become a critical challenge in data storage [1,2,3,4]. Compared to traditional methods, DNA storage technology offers superior capacity, enhanced stability, and reduced maintenance costs, positioning it as a prominent focus of contemporary information storage research [5,6,7,8].

In DNA storage systems, all files are encoded into DNA sequences composed of the four bases A, T, C, and G. These sequences are then synthesized and stored in a DNA file pool. To retrieve the desired files from the DNA file pool, PCR amplification technology is typically employed for information retrieval [9,10]. Each DNA data file contains a sequence that binds to a specific PCR primer. To extract a particular file, the corresponding primer is added to the sample to locate and amplify the desired sequence [11,12,13,14,15,16,17]. However, the binding of primers to DNA sequences is governed by the principle of base complementarity. If other base segments within the DNA sequences can form complementary pairs with the primer, it will lead to incomplete amplification of the target sequence. More critically, if other DNA sequences in the file pool form complementary pairs with the primer, cross-talk between the primer and DNA sequences will occur, resulting in the retrieval of unintended files [18,19]. The phenomenon where DNA sequences form complementary base pairs with the primer at incorrect positions is known as “nonspecific pairing”.

Due to the significant length of DNA sequences compared to primer sequences, it is nearly impossible to entirely avoid complementary pairing between base segments and the primer. Kayama et al. utilized recurrent neural networks to predict the success rate of PCR amplification for specific primer sets and DNA sequences [20]. Their experimental results indicated that when a continuous base complementarity exists at the DNA sequence and 3’ end of the primer, PCR amplification may initiate at that position, leading to nonspecific pairing errors. We conducted further experimental analysis to investigate the number of continuous complementary bases required for an increase in PCR amplification error rates. The results demonstrated that when the DNA sequence and the primer’s 3’ end exhibit eight or more consecutive complementary bases, nonspecific pairing is more likely to occur (detailed experiments and results are provided in the Supplementary Materials: S1.2. Nonspecific Pairing Constraint). Since PCR amplification involves a double-stranded reaction, if one strand of the DNA sequence contains base segments that repeat the primer, it implies that the other strand will form complementary base pairs with the primer. Therefore, to avoid nonspecific pairing between the DNA sequence and the primer, it is sufficient to ensure that the DNA sequence does not exhibit eight consecutive complementary bases with the primer’s 3’ end. We summarize this as the “nonspecific pairing constraint”.

Additionally, due to the biochemical properties of DNA sequences, they must also satisfy traditional biological constraints (detailed information is provided in the Supplementary Materials: S1.1. Conventional Biological Constraints) [21,22,23,24,25,26]. To ensure that the DNA sequences meet both constraints and to enhance the accuracy of PCR amplification, we propose an efficient DNA coding algorithm for PCR amplification information retrieval. This algorithm employs pruning optimization and variable-length scanning to adaptively establish a file coding codebook with the maximum theoretical storage density while adhering to traditional biological constraints. A codeword search tree is constructed based on the statistical characteristics of primers to optimize the codebook and minimize nonspecific pairing. Additionally, the variable-length interleaver, based on primers, interleaves the DNA sequence to further ensure compliance with biological constraints and reduce nonspecific pairing. This algorithm not only effectively addresses the problem of nonspecific pairing but also enhances storage density and reduces the cost of DNA synthesis. Furthermore, the algorithm is universal and suitable for efficient coding of files in arbitrary formats.

## 2. Results

To evaluate the performance of the proposed algorithm, we conducted both coding and simulation experiments to compare it with other coding algorithms across three dimensions: nonspecific pairing, biological constraints, and storage density. In the coding experiment, a diverse set of files was selected, including four texts, three images, one compressed package, and video and audio files. Detailed information about the experimental files is presented in Supplementary Materials: S2. Experiment Files Specific Information.

### 2.1. Experiments with Nonspecific Pairing Constraint

**Figure 1:**
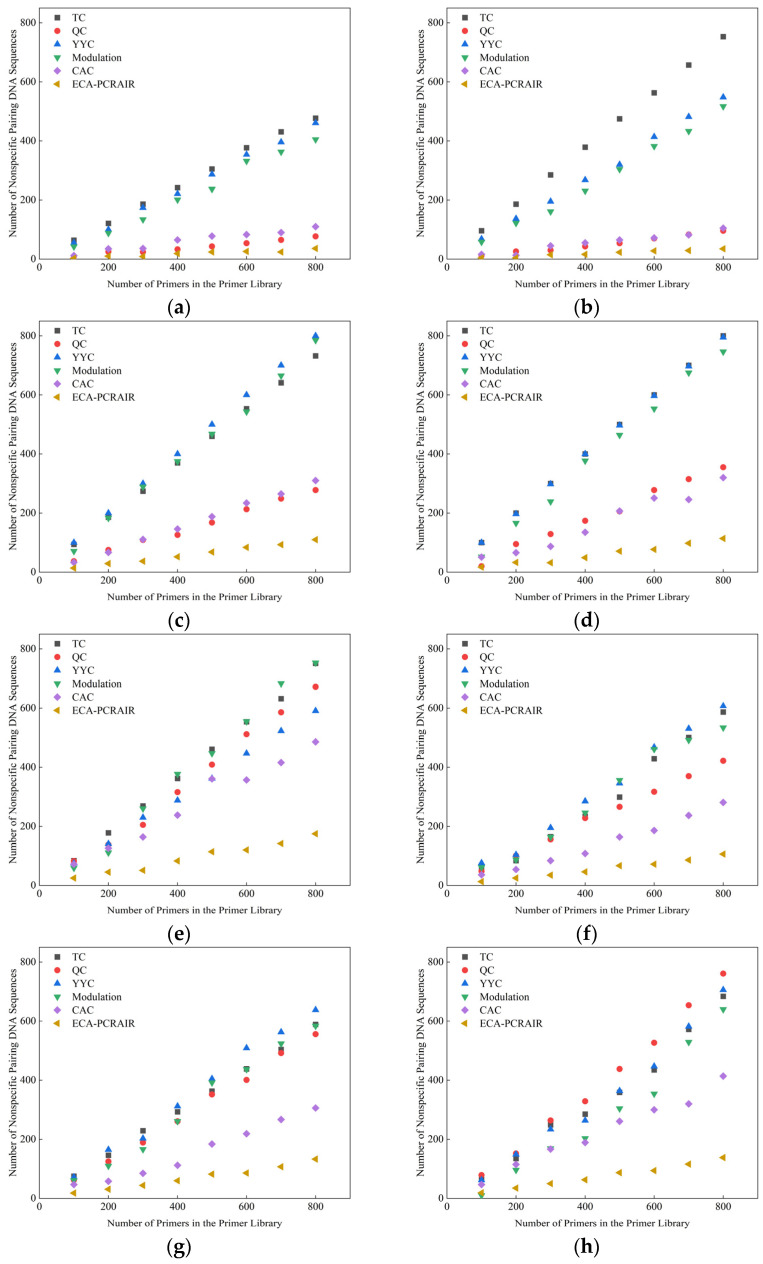

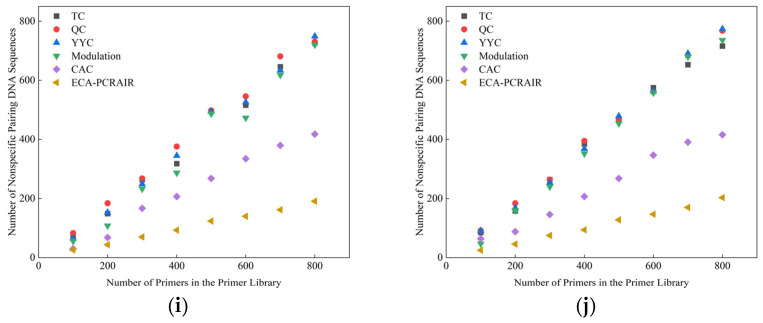
Comparison of the number of nonspecific pairing between DNA sequences and primers under different coding algorithms: (**a**) English Text 1; (**b**) English Text 2; (**c**) Chinese Text 1; (**d**) Chinese Text 2; (**e**) Color Image; (**f**) Black-and-white Image; (**g**) Gray-scale Image; (**h**) ZIP File; (**i**) MP3 File; (**j**) MP4 File.

To verify the effectiveness of the ECA-PCRAIR algorithm in addressing the nonspecific pairing constraint, we compared it against traditional TC [27], QC [28] algorithms, and the latest YYC [29], Modulation [30], and CAC [19] algorithms. We analyzed the frequency of consecutive 8-base nonspecific pairing between DNA sequences of different experimental files and the 3’ ends of primers under various coding algorithms. The experimental results are illustrated in Figure 1.

**Figure 2:**
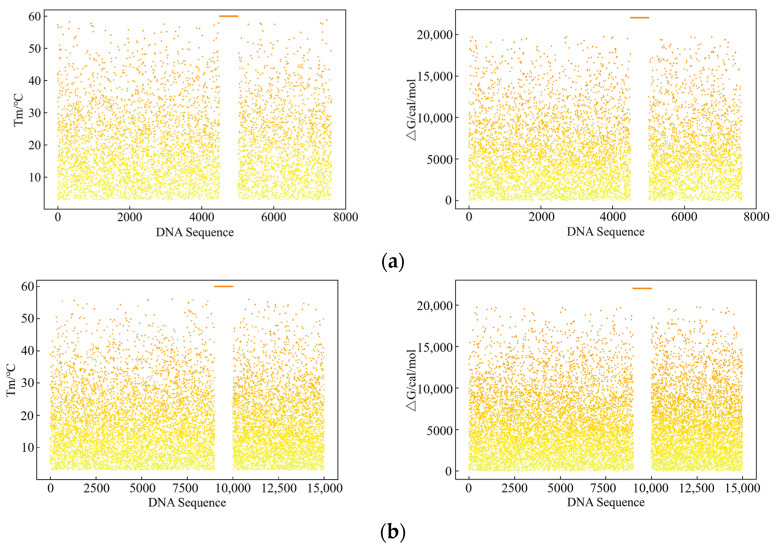

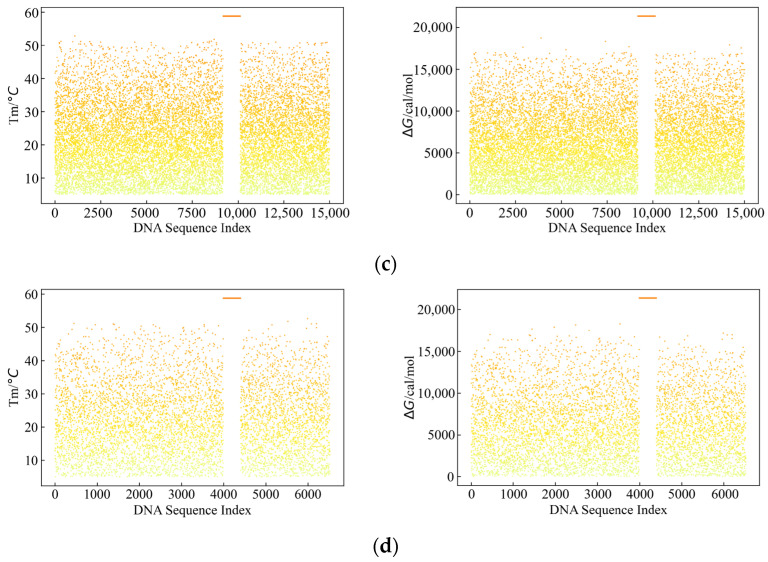
Tm and ΔG simulation results of DNA sequences with different coding algorithms: (**a**) YYC; (**b**) Modulation; (**c**) CAC; (**d**) ECA-PCRAIR.

Additionally, we coded 16 image files into DNA sequences using the YYC, Modulation, CAC, and ECA-PCRAIR algorithms, resulting in final sequence counts of 7600, 15,000, 15,000, and 6522, respectively. Tm and ΔG were used as indicators for comparative experiments. The upstream primer used in this experiment was “AATTGACGTATTGCTCGACC”. We conducted a thermodynamic analysis of the primer sequence and the four sets of DNA sequences, examining the melting temperatures Tm and ΔG between the primer and each DNA sequence. The results are presented in Figure 2.

### 2.2. GC Content and Homopolymer Condition

**Figure 3:**
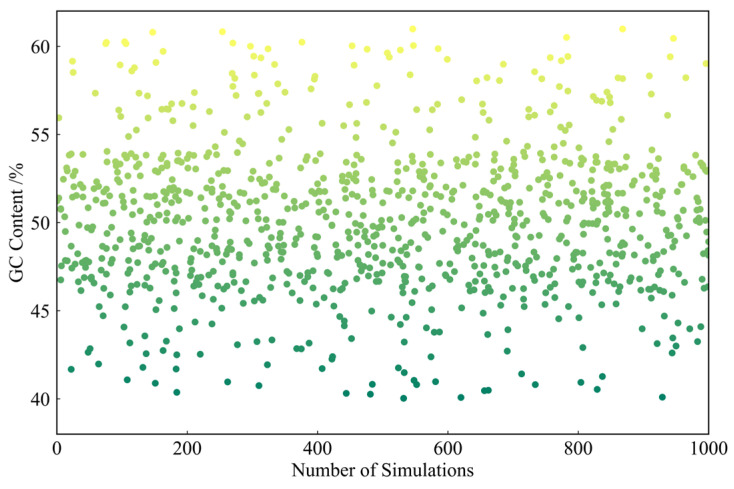
GC content after coding 1000 random sequences.

**Figure 4:**
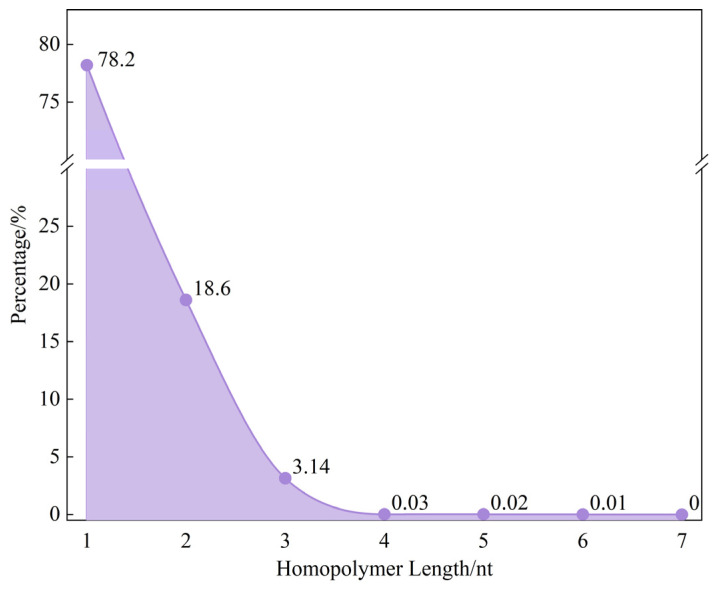
Percentage of homopolymer after coding 1000 random sequences.

To evaluate the performance of the ECA-PCRAIR algorithm under traditional biological constraints, both coding and simulation experiments were conducted. The coding experiments are presented in the Supplementary Materials “3. GC Content and Homopolymer Coding Experiments”. In the simulation experiments, we randomly generated 1000 binary code streams, each 1000 bits in length, and coded them using the ECA-PCRAIR algorithm. The GC content and homopolymer occurrences of the DNA sequences were counted. The GC content of the 1000 sequences is shown in Figure 3, while the homopolymer data is presented in Figure 4.

### 2.3. Coding Performance Experiment

**Figure 5:**
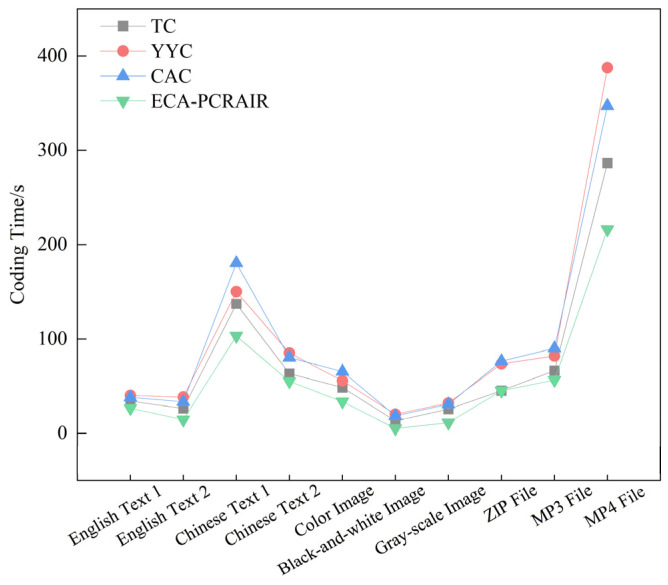
Comparison of coding times for different files under TC, YYC, CAC and ECA-PCRAIR algorithms.

To compare the coding rate of the proposed algorithm, we measured the time required by the ECA-PCRAIR algorithm and compared it with that of the TC, YYC, and CAC algorithms. The experimental results are shown in Figure 5.

**Figure 6:**
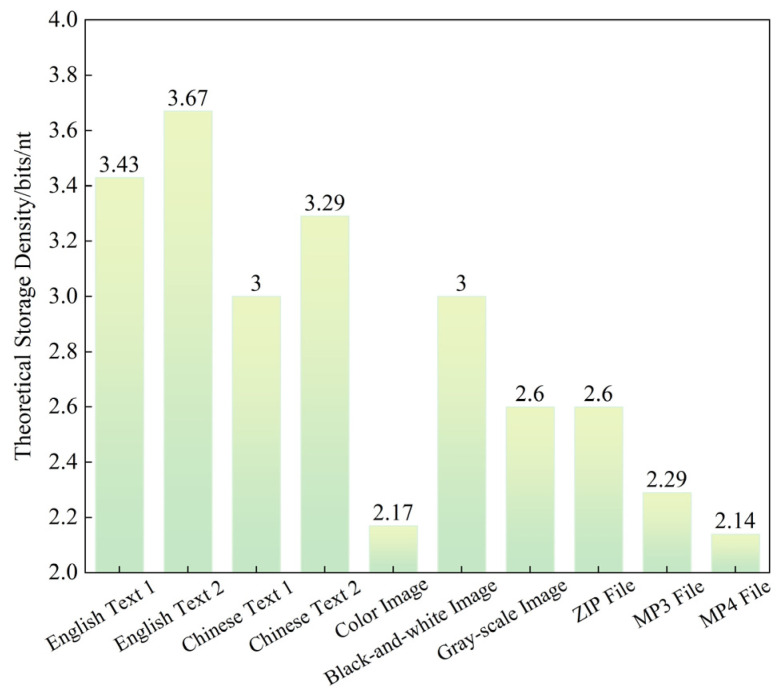
Theoretical storage density for different files under the ECA-PCRAIR algorithm.

The theoretical storage density of the ECA-PCRAIR algorithm for different files is summarized in Figure 6.

## 3. Discussion

The experimental results demonstrate that the probability of nonspecific pairing between the DNA sequence and the 3’ end of the primer in the traditional QC algorithm ranges from 10% to 76%, while in the TC algorithm, it is between 71% and 99%. For the YYC, Modulation, and CAC algorithms, the probabilities of nonspecific pairing are 64% to 94%, 10% to 98%, and 7% to 72%, respectively. The ECA-PCRAIR algorithm exhibits a significantly lower nonspecific pairing probability, ranging from 2% to 25%. This substantial reduction highlights the superior ability of the ECA-PCRAIR algorithm in mitigating nonspecific pairing compared to the other five algorithms, thereby indicating its exceptional effectiveness in addressing the nonspecific pairing constraint.

Due to the inevitable nonspecific pairing under the YYC, Modulation, and CAC algorithms, the Tm and ΔG values of the DNA sequences are very close to those of the given primer. Consequently, when the target primer is used to retrieve the target file from the DNA file pool, non-target files are also erroneously retrieved, complicating subsequent decoding and causing PCR amplification bias. In contrast, the DNA sequences coded by the ECA-PCRAIR algorithm exhibit almost no nonspecific pairing. As shown in Figure 2d, the thermodynamic indices of other unrelated file sequences, whose Tm and ΔG values are close to the target file, diverge significantly after being processed by the ECA-PCRAIR algorithm. This makes it difficult to amplify files other than the specific target in PCR amplification experiments, resulting in more accurate target amplification and ideal experimental outcomes. Thus, the ECA-PCRAIR algorithm effectively avoids the nonspecific pairing problem between DNA sequences and the primer’s 3’ end, enhancing the accuracy of PCR amplification and the proportion of effective information.

The demonstrated effectiveness of the ECA-PCRAIR algorithm can be attributed to its innovative coding process, which leverages a codeword search tree derived from the primer library. The algorithm constructs the codebook based on the ascending order of codeword weights, preferentially selecting base combinations that either do not appear in the primer or have a low probability of occurrence. This strategy effectively minimizes nonspecific pairing between the DNA sequence and the primer. In contrast, other algorithms primarily focus on traditional biological constraints and storage density without systematically addressing the nonspecific pairing between DNA sequences and primers. Given the vast number of potential DNA sequences and the limited number of primers in a DNA file pool, nonspecific pairing with primers becomes inevitable. Consequently, existing coding algorithms fail to fully satisfy the nonspecific pairing constraints. The CAC algorithm partially addresses nonspecific pairing. However, it is fundamentally a coding scheme based on primer control construction. It only ensures that the DNA sequence does not exhibit nonspecific pairing with a specific primer. In practical applications, where hundreds or thousands of primers may be present, DNA sequences are prone to nonspecific pairing with other primers. Therefore, the Tm and ΔG values of CAC are higher than those of the proposed ECA-PCRAIR algorithm, and its binding force is inferior to our algorithm, as shown in Figure 2c. Furthermore, the theoretical storage density of CAC is limited to 1 bit/nt, significantly lower than that of the ECA-PCRAIR algorithm. In conclusion, the overall performance of the CAC algorithm is inferior to that of the ECA-PCRAIR algorithm.

Based on empirical data from file and simulation experiments, the ECA-PCRAIR algorithm effectively manages GC content and homopolymer situations. The experiments demonstrated that GC content was consistently maintained between 40% and 60%, with a concentration range of 45% to 55%. Long homopolymers were kept below 4 nt, and homopolymers of 4 nt or longer appeared in only 0.06% of cases. This minimal occurrence is unlikely to substantially impact the overall PCR reaction, underscoring the ECA-PCRAIR algorithm’s superior performance in controlling GC content and homopolymer formation.

Compared with the TC, YYC, and CAC algorithms, the ECA-PCRAIR algorithm exhibits a faster coding speed and reduced coding time for various file formats. The coding time is directly proportional to the file size; larger files necessitate longer coding time. For text files, the ECA-PCRAIR algorithm reduces time consumption by an average of 29% compared to the TC, YYC, and CAC algorithms. For image files, the reduction averages 44%, highlighting the most significant performance improvement. For other file formats, time consumption decreases by approximately 26% on average. This improvement is attributable to the low time complexity of the ECA-PCRAIR algorithm, with the primary computational efforts concentrated in variable-length scanning and search tree weighting, making the computational complexity linear with the input data size. Once these components are completed, the subsequent coding involves straightforward codebook mapping. In contrast, the TC, YYC, and CAC algorithms require iterative comparisons with previous bases during coding, leading to decreased efficiency as the DNA sequence lengthens and the number of iterations increases. Consequently, these algorithms demand more computational resources. Among these, the ECA-PCRAIR algorithm shows the highest reduction rate in time consumption for image files due to the regularity of the binary code streams in such files. The text and other file formats, being more complex, exhibit less regular binary data patterns. The core principle of the ECA-PCRAIR algorithm is to identify combinatorial rules within binary code streams to derive the most efficient codebook. Therefore, more regular binary data results in reduced time consumption for the ECA-PCRAIR algorithm.

The ECA-PCRAIR algorithm adaptively identifies the coding method with the highest storage density for different files. Its theoretical storage density ranges from 2.14 to 3.67 bits/nt, as depicted in Figure 6. In comparison, current coding algorithms achieve a theoretical density of 1 to 1.98 bits/nt [19,27,28,29,30,31,32,33,34,35,36,37,38,39]. This demonstrates that the proposed algorithm significantly enhances storage capacity, thereby reducing the cost of DNA sequence synthesis and accelerating the adoption and application of DNA storage technology.

## 4. Methods

**Figure 7:**
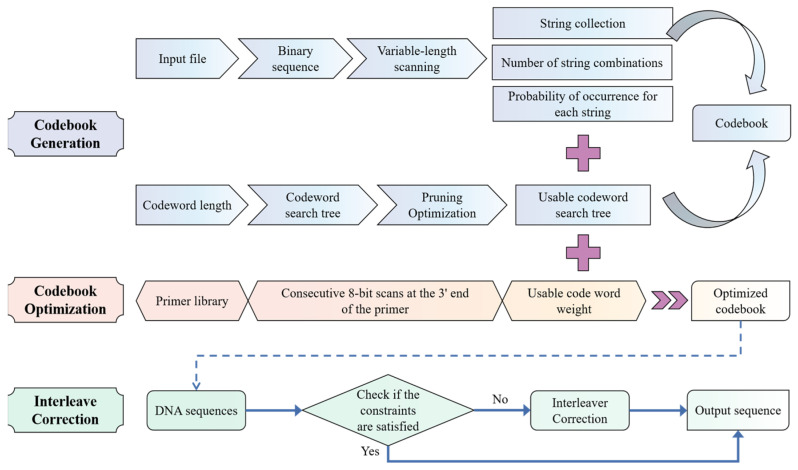
Schematic diagram of the flow of the ECA-PCRAIR algorithm.

The proposed ECA-PCRAIR algorithm comprises three main steps: codebook generation, codebook optimization, and interleaved correction. By analyzing the statistical characteristics of the binary bit stream of the file to be coded, the initial codebook is generated. This initial codebook is then optimized by incorporating the nonspecific pairing constraint to produce the final codebook for the file. Finally, through the interleaving algorithm, the constraint conditions of the coded DNA file sequence are detected and corrected, resulting in a set of file sequences that can be directly synthesized by DNA molecules. A schematic of the coding algorithm is shown in Figure 7.

### 4.1. Codebook Generation Algorithm Based on Pruning Optimization and Variable-Length Scanning

#### 4.1.1. Codeword Generation Algorithm Based on Pruning Optimization

For a DNA sequence of length N, the space of alternative bases at each position is ℝ={A,T,C,G} and its size is sizeof(ℝ)=4. Therefore, there are at most $4^{N}$ possibilities for a sequence of length N. However, a significant portion of these possible sequences cannot be used in practice. Sequences with a high number of consecutive repeated base subsequences, GC content lower than 40% or higher than 60%, or complementary regions within different parts of the sequences that lead to hairpin structures can significantly affect the accuracy of DNA molecule synthesis, selective PCR amplification reactions, and DNA molecule sequencing. Thus, it is necessary to filter the sequences according to these constraints to obtain viable codewords for each length.

Firstly, the length of the base sequence N is determined, and a multi-branch search tree with depth N is established by taking the optional base space ℝ={A,T,C,G} at each position as the root node. Each search tree has a total of $3^{N}$ different combination patterns, and there are a total of $4^{N}$ species.

After constructing the search tree, a depth-first traversal of the tree nodes is performed. Starting from the root node, child nodes are visited depth-first to determine whether the biological constraints on homopolymer length and GC content are met. If the visited node does not meet the constraint conditions, the current search path is marked, and the search tree is pruned to optimize the time complexity and space complexity of the search algorithm.

**Figure 8:**
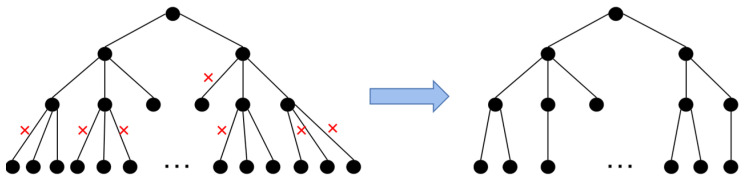
Search tree generated based on the pruning optimization algorithm.

These steps of child node visitation, biological constraints judgment of node path base sequences, and pruning optimization are repeated until all feasible paths have been visited. The remaining node paths of the search tree, after pruning optimization, represent all the alternative codewords that meet the basic biological constraint conditions under fixed length, as depicted in Figure 8.

After the aforementioned search process, a feasible codebook consisting of codewords of length N is obtained. By altering the codeword length N and repeating the above steps, a set of feasible codeword sets and the relationship between the length of the codeword N and the number of alternative codewords are established for the given orthogonal primer library. Typically, the constraints for the codeword search and pruning process are homopolymer length <4 nt and GC content between 40% and 60%.

#### 4.1.2. Codebook Generation Algorithm Based on Variable-Length Scanning

Considering that different input files have varying statistical characteristics in their code streams, we scan the input files with variable lengths. By comparing the compression efficiency of the scan results for different lengths, we select the optimal scan length parameter and then use the pruning optimization algorithm to match the best codeword length, thereby obtaining the adaptive codebook. The adaptive codebook is generated as follows.

For a binary sequence of length $L_{0}$, given the string length $l_{0}$. The binary sequence is complemented with “0” according to $l_{0}$ to obtain the new sequence and its length $L^{*}$. $L_{0}$ and $L^{*}$ should satisfy the following relationship:

(1) $$L^{*}=\left\{ \begin{array}{l} L_{0},\;\;\;\;\;\;\;if\;L_{0}\mathrm{mod}l_{0}=0\\ L_{0}+(l_{0}-L_{0}\mathrm{mod}l_{0}),\;else \end{array}\right.$$

String sets $S=\{s_{1},s_{2},s_{3},\cdots ,s_{q}\}$ can be obtained by scanning new binary sequences with $l_{0}$ bit intervals. In different sets of strings, each string has a different probability of appearing. Let the probability of each character be $p_{i}$, then:

(2) $$p_{i}=P(s_{i}),$$

among them,

(3) $$0\leq p_{i}\leq 1,\;\sum_{i=1}^{q}p_{i}=1$$

For the string set S obtained from the binary sequence after the interval scanning, the codebook is C and the code length is $l_{1},l_{2},\ldots ,l_{q}$. The average code length is:

(4) $$L=L(C)=\sum_{i=1}^{q}p_{i}l_{i}$$

Using different length interval $l_{0}$ for scanning, the corresponding average code length can be obtained. From the perspective of coding efficiency and DNA storage cost, the smaller the average code length, the better. Considering that different texts have different statistical characteristics, the optimal scan length should be selected according to the characteristics of the text.

The steps for the best code length matching are as follows:

(1) An initialized numeric value $l_{0}$ (usually $l_{0}=3$) is selected, and a new binary stream is obtained by adding “0” to the original binary sequence. The probability distribution of the string with length $l_{0}$ in the binary stream is counted, and the required codeword length $l_{i}$ is matched according to the pruning optimization algorithm.

(2) Calculate the theoretical storage density D of a single codeword according to the string bits $l_{0}$ and the code length $l_{i}$ obtained by matching. Record the string bits $l_{0}$, the codeword length $l_{i}$ and the theoretical storage density D. However, since the code length allocation algorithm will produce a case where one file corresponds to one codebook, the length of the codebook should be added when calculating the actual theoretical storage density D. The formula is as follows:

(5) $$D=\frac{L^{\prime }+L_{c}}{L_{0}}=\frac{\sum_{i=1}^{q}\frac{L^{*}}{l_{0}}\cdot p_{i}\cdot l_{i}+\sum_{i=1}^{q}\left(l_{0}+l_{i}\right)}{L_{0}},$$

which $L^{\prime }$ denotes the length of the coded base sequence and $L_{c}$ denotes the codebook length.

(3) Let $l_{0}=l_{0}+1$, repeat the above steps, and finally obtain the one-to-one dictionary Z of $l_{0}$, $l_{i}$ and D. It should be noted that the number of scanning bits cannot be increased infinitely. Based on experimental experience, the upper limit of scanning bits N=30 is set.

(4) Sort the dictionary Z in descending order according to the theoretical storage density D, and select the string length $l_{0}$ and the codeword length $l_{i}$ with the highest theoretical storage density.

After the optimal code length matching, the optimal string length and codeword length have been obtained, based on which the initial adaptive coding codebook can be generated.

### 4.2. Codebook Optimization Algorithm Based on Nonspecific Pairing Constraint

According to the codebook generation algorithm described in the previous section, the parameters for the codebook that achieves optimal theoretical storage density have been determined. These parameters include the number of string bits, the codeword length, the search tree at this codeword length, and the initial codebook. Although the initial codebook generated by the algorithm provides the best information storage density, it has two main limitations: (1) the mapping relationship between strings and codewords in the codebook has not been constrained, and (2) the nonspecific pairing constraints of DNA storage systems have not been considered during the codebook generation process. Consequently, the initial codebook requires optimization.

Upon further investigation, it was observed that the base distribution of primer sequences in the primer library exhibits specific statistical characteristics and is not uniformly distributed. Detailed experimental procedures are provided in the Supplementary Materials “4. Analysis of Primer Statistical Properties”. Considering this feature, we propose a codebook optimization algorithm based on nonspecific pairing constraints to enhance the initial codebook. By leveraging the statistical characteristics of the base substrings in the primer library, we assign a weight value to each node of the codeword search tree obtained previously. Based on these weight values and the frequency of the strings to be coded, we map the codewords to the strings, thereby optimizing the codebook and completing the preliminary coding. The main steps are as follows:

(1) Sliding Scan: Perform an $l_{i}$-bit sliding scan on the last 8 bases of all primers in the primer library. After each scan, the path weight w←w+1 of the corresponding codeword is updated according to the scanning results on the $l_{i}$ bit search tree obtained by the pruning optimization algorithm. The result is an $l_{i}$-bit search tree with path weights.

(2) Mapping: Starting from the path with the least sum of path weights, perform a one-to-one mapping using the $l_{0}$-bit string determined in the variable length scan, according to the string occurrence probability from high to low. This mapping method ensures that the continuous 8-base fragment at the 3’ end of the primer appears as infrequently as possible in the coding sequence, thereby reducing the likelihood of nonspecific pairing between the DNA sequence and the 3’ end of the primer library.

### 4.3. Variable-Length Interleaving Models with Nonspecific Pairing Constraint

After the codebook optimization process, the probability of nonspecific pairing between the DNA sequence and the 3’ end of primers in the orthogonal primer library is greatly reduced. However, due to the large number of DNA molecules storing information in each DNA file pool, the number of different file DNA sequences usually exceeds 10^4^. During the conversion of binary information sequences into DNA sequences, according to the codebook, the connection between different codewords can still cause some nonspecific pairing issues between partial string positions of the DNA sequence and the 3’ end of the primer sequence.

To address this problem, we design a variable-length interleaver. By establishing a criterion to determine whether the load part of the DNA sequence satisfies the nonspecific pairing constraint and other biological constraints, DNA sequences that do not meet these criteria are input into the variable-length interleaver for cyclic interleaving calibration, transforming them into risk-free sequences.

**Figure 9:**
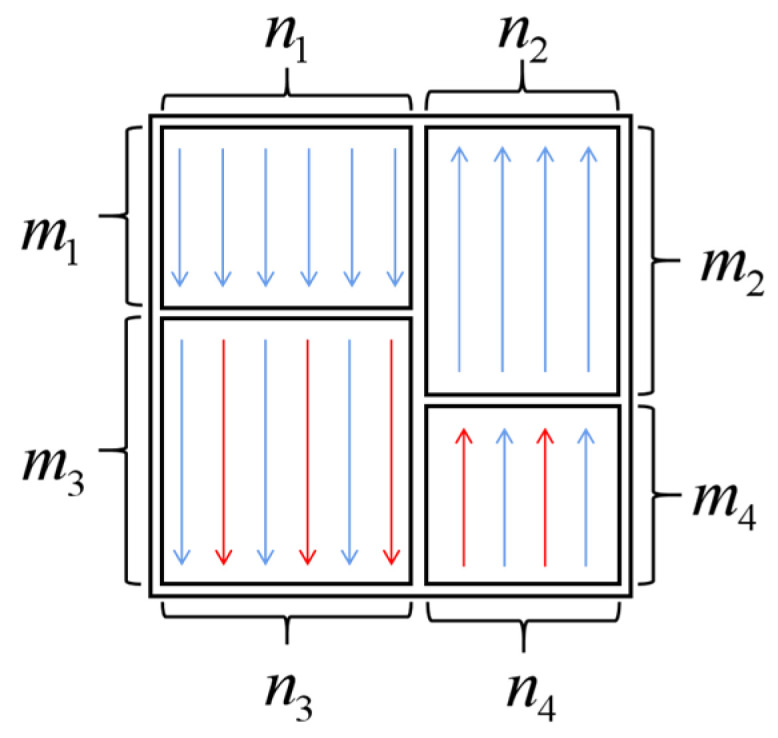
Diagram of the variable-length adaptive packet interleaver.

Figure 9 shows an example diagram of the variable-length adaptive packet interleaver, where the existing interleaver is divided into four blocks with different sizes, and each sub-block has different interleaving criteria.

The overall interleaver size N×M will adapt to the length of the input sequence L by adjusting to square interleavers, calculated as follows:

(6) $$\min\;Diff_{0}=n-m|N\times M=L\;\;\;\;s.t.N\neq M$$

Simultaneously, the interleaver is adaptively divided into four groups of smaller interleavers of different sizes, tending towards square shapes. The size of the 1 interleaver is specified as $n_{1}\times m_{1}$, the 2 interleaver is specified as $n_{2}\times m_{2}$, the 3 interleaver is specified as $n_{3}\times m_{3}$, and the 4 interleaver is specified as $n_{4}\times m_{4}$. The dimensions of the four interleaver groups are calculated as follows.

(7) $$\min\;Diff_{1}=n_{1}-n_{2}|n_{1}+n_{2}=N\;\;\;\;s.t.n_{1}\neq n_{2}$$

(8) $$\min\;Diff_{2}=m_{1}-m_{2}|m_{1}+m_{2}=M\;\;\;\;s.t.m_{1}\neq m_{2}$$

(9) $$n_{3}=n_{1},\;n_{4}=n_{2}$$

(10) $$m_{4}=m_{1},\;m_{3}=m_{2}$$

After each interleaving operation, the DNA sequence is evaluated to determine if it still poses a risk. If nonspecific pairing remains a concern, the interleaving process is repeated. Otherwise, the risk-free sequence is output. By using the variable-length adaptive group interleaver, risk-free sequences that satisfy all biological constraints can be generated through base rearrangement without altering the types, numbers, and GC content of the original DNA base sequences.

## 5. Conclusions

In PCR amplification reactions, nonspecific pairing between base segments of the DNA sequence and the primer, particularly when it involves eight or more consecutive bases, can easily cause cross-talk in the amplification process. This leads to the generation of redundant sequences and a reduction in amplification accuracy. To address this issue, we propose a novel DNA coding algorithm tailored for PCR amplification information retrieval. This algorithm constructs a weighted codeword search tree based on a primer library and codes with base combinations that are either absent or have a low occurrence probability in the primer library. Additionally, it employs a variable-length interleaver for constraint detection and correction, significantly reducing nonspecific pairing between DNA sequences and primers. The preliminary codebook generation process incorporates pruning optimization and variable-length scanning, which not only ensures the satisfaction of traditional biological constraints but also adaptively searches for the optimal coding scheme in terms of storage density, thereby enhancing storage capacity.

Experimental results demonstrate that the DNA sequences coded by our proposed algorithm exhibit a nonspecific pairing probability with primers of only 2% to 25%, which is significantly lower than that of existing algorithms. Furthermore, the theoretical storage density of our algorithm reaches 2.14 to 3.67 bits/nt, more than twice that of current algorithms. This algorithm holds significant potential for advancing the practical application of DNA storage.

## Data Availability

The data presented in this study are available on request from the corresponding author.

## Supplementary Materials

The following supporting information can be downloaded at https://github.com/sunny979/-Supplementary-Materials-for-IJMS.
